# The role of silicon in plant tissue culture

**DOI:** 10.3389/fpls.2014.00571

**Published:** 2014-10-21

**Authors:** Iyyakkannu Sivanesan, Se Won Park

**Affiliations:** Department of Molecular Biotechnology, Konkuk UniversitySeoul, South Korea

**Keywords:** acclimatization, epicuticular wax deposition, hyperhydricity, organogenesis, silicon, stress tolerance

## Abstract

Growth and morphogenesis of *in vitro* cultures of plant cells, tissues, and organs are greatly influenced by the composition of the culture medium. Mineral nutrients are necessary for the growth and development of plants. Several morpho-physiological disorders such as hooked leaves, hyperhydricity, fasciation, and shoot tip necrosis are often associated with the concentration of inorganic nutrient in the tissue culture medium. Silicon (Si) is the most abundant mineral element in the soil. The application of Si has been demonstrated to be beneficial for growth, development and yield of various plants and to alleviate various stresses including nutrient imbalance. Addition of Si to the tissue culture medium improves organogenesis, embryogenesis, growth traits, morphological, anatomical, and physiological characteristics of leaves, enhances tolerance to low temperature and salinity, protects cells and against metal toxicity, prevents oxidative phenolic browning and reduces the incidence of hyperhydricity in various plants. Therefore, Si possesses considerable potential for application in a wide range of plant tissue culture studies such as cryopreservation, organogenesis, micropropagation, somatic embryogenesis and secondary metabolites production.

## INTRODUCTION

Plant tissue culture is a collection of experimental procedures for aseptic culture of isolated plant cells, tissues or organs on nutrient media under controlled environmental conditions. Growth and morphogenesis of *in vitro* cultures of plant cells, tissues and organs are greatly influenced by the composition of the culture medium. The composition of a culture medium has often been modified to stimulate the growth of particular plant material. In general, plant tissue culture medium composed of inorganic nutrients, organic supplements, carbon source, plant growth regulators and a solidifying agent. Mineral nutrients are necessary for growth and development of plants. The optimization of inorganic nutrients in the culture medium improves growth and morphogenesis of plant cells, tissues and organs *in vitro*. Several physiological disorders such as hooked leaves, hyperhydricity, fasciation and shoot tip necrosis are often associated with the concentration of inorganic nutrients in the culture medium (Reed et al., 2013).

Silicon (Si) is the most abundant mineral element in the soil (Epstein, 1999). Numerous studies have shown that Si treatment improves the growth and yield of various plants, particularly when they are subjected to both abiotic and biotic stresses (Ma, 2004). Several researchers have reviewed the role Si on plant tolerance to abiotic (Balakhnina and Borkowska, 2013; Zhu and Gong, 2014) and biotic stresses (Van Bockhaven et al., 2013). The availability of Si in hydroponic and substrate plant production system is restricted. Addition of Si to the nutrient solution or soilless substrate enhanced growth traits, yield and quality of several crops (Voogt and Sonneveld, 2001). The promoting effects of Si on plants might be due to increasing nutrient uptake and photosynthetic activity. Though Si is a ubiquitous contaminant, the use of silicon-free containers and double distilled water restricts its availability. Furthermore, Si has not been included in any commercial tissue culture media formulation. The inclusion of Si to the culture medium improved the morphogenetic potential of plant cells, tissues and organs. Several studies have shown that the inclusion of Si to the tissue culture medium enhances callus growth, shoot regeneration, and root induction and stimulates somatic embryogenesis, and improve morphological, anatomical and physiological characteristics of plantlets. In addition, Si treatment prolongs the longevity of calli and organs with a potential for plant regeneration. The inclusion of Si to the tissue culture medium also enhances tolerance to low temperature, metal toxicity and salinity. Si enhancing tolerance of plants to various stresses by altering activity of antioxidant enzymes, cation binding capacity of the cell walls, endogenous plant hormone level, increasing production of chitinase, glucanse, lignin, phenolics, and phytoalexins, nutrient uptake, improving strength of cell and plant, maintaining the structure of stomata, relative water content, and reducing uptake of heavy metals. This review concentrates the potential roles of Si in plant tissue culture.

## ROLE OF SI IN PLANT TISSUE CULTURE

### ORGANOGENESIS AND SOMATIC EMBRYOGENESIS

Islam et al. (2005) investigated the effect of calcium silicate (CaSiO_3_) on callus induction and plant regeneration from mature seed explants of rice ‘Kalizira’, ‘Lucky’, and ‘Pajam’. The highest frequency of callus induction is achieved on Murashige and Skoog (MS) medium containing CaSiO_3_. However, plant or root regeneration potential of rice calli is cultivar depended. Similarly, effects of Si on plant or root development depend on reed (*Phragmites australis*) genotype used for callus induction (Mathe et al., 2012). Addition of Si as sodium silicate (Na_2_SiO_3_) to the modified MS medium promotes the growth of calli obtained from stem nodal and root explants of *P. australis* while its effect on somatic embryogenesis is explants dependent: it stimulates embryogenesis of root calli, but it does not influence this process in stem nodal calli. Soares et al. (2011) evaluated the effect of Si source [potassium silicate (K_2_SiO_3_) and Na_2_SiO_3_] on shoot multiplication of *Cattleya loddigesii*. The highest number of shoots is observed on the modified Knudson C medium containing 5.0 mg L^-1^ K_2_SiO_3_. In *Ajuga multiflora*, addition of Si to MS medium containing 2iP and IAA, enhanced adventitious shoot regeneration (about threefold) by increasing the activity of antioxidant enzymes such as SOD, POD, APX, and CAT (Sivanesan and Jeong, 2014). In addition, the authors observed the Si accumulation in leaves of plants developed in the culture medium with Si, but not in plants developed in the medium without Si by wavelength dispersive *X*-ray analysis. These studies indicate that the effect of Si on morphogenetic potential of *in vitro* plant cultures depends on plant species, genotype and concentration of Si in the culture medium. Still further studies are required to better understand the biochemical and molecular mechanism of Si on organogenesis and somatic embryogenesis.

## GROWTH AND DEVELOPMENT

The application of Si has been reported to enhance the growth and development of various plants. Zhou (1995) observed silica bodies in leaf tissues of *Phalaenopsis* hybrid plantlets grown in Vacin and Went medium supplemented with CaSiO_3_. Addition of CaSiO_3_ also increased the leaf length. Soares et al. (2011) reported that the addition of 5.0 mg L^-1^ K_2_SiO_3_ and 20.0 mg L^-1^ Na_2_SiO_3_ to the modified Knudson C medium increased the number of roots and length of aerial part and root in seedlings of *C. loddigesii*. Subsequently the same group investigated the effect of GA_3_ and Na_2_SiO_3_ on growth and development of *C. loddigesii* (Soares et al., 2013). It was reported that the combination of GA_3_ and Na_2_SiO_3_ increased the number of leaves and roots than GA_3_ alone. The optimal concentration of Si varies within the same plant species and or genotype. The inclusion of CaSiO_3_ at 0.5 and 2.0 mg L^-1^ to the MS medium stimulates the growth of native (*Brassavolva perrinii*) and hybrid (*Laelia cattleya* ‘Culminant Tuilerie’ ×*L. cattleya* ‘Sons Atout Rotunda’) × *Brassolaelia cattleya* ‘Startifire Moon Beach’) orchid plants, respectively (Soares et al., 2012). Lim et al. (2012) also reported that the effect of Si (K_2_SiO_3_) on the growth traits of begonia ‘Super Olympia Red’ and ‘Super Olympia Rose’ and pansy ‘Matrix White Blotch’ and ‘Matrix Yellow Blotch’ are mainly dependent on the cultivars. Braga et al. (2009) investigated the effect of different Si sources such as CaSiO_3_, K_2_SiO_3_, and Na_2_SiO_3_ on the growth and anatomical characteristics of strawberry ‘Oso Grande’ seedlings. The fresh and dry weight of seedlings increased in MS medium containing 1.0 g L^-1^ Na_2_SiO_3_. Seedlings of banana ‘Maca’ cultured in the medium supplemented with CaSiO_3_ increased the chlorophyll content, whereas those cultured in the medium containing Na_2_SiO_3_ increased length, fresh and dry weight of shoots (Asmar et al., 2011).

The morphological and anatomical characteristics of *in vitro*-grown plantlets are different from the field-grown seedlings. Si inclusion to the rooting medium increased leaf tissue thickness and epicuticular wax deposition in banana (Asmar et al., 2013a) and strawberry (Braga et al., 2009) plantlets. Luz et al. (2012) reported that supplementation of CaSiO_3_, K_2_SiO_3_, or Na_2_SiO_3_ to the rooting medium improved leaf anatomy of banana ‘Maca’ plantlets. The inclusion of CaSiO_3_ to the culture medium also increased photosynthetic rate and chlorophyll content of banana plantlets (Asmar et al., 2013b). In strawberry, light and electron microscopic analysis showed deformation in chlorenchyma and the epidermis of leaves from plantlets grown in the culture medium devoid of Si (Soares et al., 2012). Recently, He et al. (2013) confirmed the deposition of Si within the cell walls of *in vitro*-cultured rice cells. Si improves the structural stability of cell walls during cell elongation and division and thereby maintained cell shape, which may be important for the function and survival of cells (**Table 1**).

**Table 1:** Role of Si in plant tissue culture.

Plant species	Role of Si	Reference
*Ajuga multiflora*	Increased frequency of shoot regeneration Increased resistance to NaCl	Sivanesan and Jeong (2014)
*Begonia semperflorens*	Increased growth, biomass and chlorophyll content	Lim et al. (2012)
*Brassavola perrinii* Hybrid orchid	Increased seedlings growth, favorable characteristics in the leaf anatomy of the orchid seedlings	Soares et al. (2012)
*Cattlleya loddigesii*	Increased the number of shoots	Soares et al. (2011)
	Increased growth traits of seedlings	Soares et al. (2013)
*Coleus hybridus*	Maintained regenerative potential of vitroplantlets	Radovet et al. (2008)
*Cotoneaster wilsonii*	Reduced hyperhydricity	Sivanesan et al. (2011)
*Dendrobium moniliforme*	Increased resistance to low temperature	Duan et al. (2013)
*Fragaria* ×*ananassa*	Increased biomass, thickness of leaf tissues and epicuticular wax deposition	Braga et al. (2009)
*Musa* sp. ‘Grande Naine’	Well-developed stomata, Increased epicuticular wax layer in leaves	Asmar et al. (2013a)
*Musa* sp. ‘Maca’ banana	Increased chlorophyll content, biomass and seedlings growth	Asmar et al. (2011)
	Improved leaf anatomy	Luz et al. (2012)
	Improved morpho-physiological leaf characteristics of seedlings	Asmar et al. (2013b)
*Ornithogalum dubium*	Reduced hyperhydricity and improving leaf structure and *ex vitro* survival during acclimatization	Ziv (2010)
*Oryza sativa*	Improved callus induction, and plant regeneration Improved structural stability of cell walls	Islam et al. (2005)


		He et al. (2013)
*Perilla frutescens*	Increased the growth rate and content of anthocyanins	Zhong et al. (1992)
*Phalaenopsis* hybrid	Increased shoot growth	Zhou (1995)
*Phragmites australis*	Stimulated callus growth, somatic embryogenesis and root formation	Mathe et al. (2012)
*Picea abies*	Ameliorated the effect of Al	Prabagar et al. (2011)
*Psidium guajava*	Inhibiting phenol-based browning	Youssef et al. (2010)
*Salvia splendens*	Increased resistance to NaCl	Soundararajan et al. (2013)
*Solanum tuberosum* var. *Gersa*	Maintained regenerative potential of vitroplantlets	Radovet-Salinschi and Cachita-Cosma (2012)
*S. tuberosum*	Increased growth, biomass and tolerance to NaCl	Qing et al. (2005)
*Viola* ×*wittrockiana*	Increased growth, biomass and chlorophyll content	Lim et al. (2012)
*Vitis vinifera* ×*V. labrusca*	Increased the survival rate of callus under low temperature	Moriguchi et al. (1988)

Ziv (2010) investigated the effect of silicon on hyperhydricity in *Ornithogalum dubium*. Addition of Na_2_SiO_3_ to MS liquid medium containing BA, NAA and 6% sucrose in bioreactors, significantly reduced induction of hyperhydric shoots, and increased plant firmness and mechanical strength. Si treatment significantly reduced the content of hydrogen peroxide and activity of oxidative reductive enzymes such as APX, ascorbate oxidase and GPX in leaves of the regenerated shoots of *O. dubium* when compared with the control (**Table 1**). Similarly, addition of Si as K_2_SiO_3_ to MS medium reduced the hyperhydricity in *Cotoneaster wilsonii* by decreasing the content of MDA in the regenerated shoots when compared with the control (Sivanesan et al., 2011). The authors observed the presence of Si in the in non-hyperhydric plants, but not in the hyperhydric leaf samples of *C. wilsonii* by energy dispersive *X*-ray analysis. Thus, the problem of hyperhydricity can be reduced by the inclusion of Si to both liquid and solid culture medium. Phenolic oxidative tissue browning is one of the bottlenecks in woody plant tissue culture. In guava, tissue browning was completely prevented by sealing the nodal explants cut ends with Si (Youssef et al., 2010) and there was no detrimental effect of Si on the subsequent steps of *in vitro* propagation. The authors suggested that Si could be used during explants preparation to control phenolic tissue browning in other plants. The morphological, anatomical and physiological characteristics of plantlets can improve *in vitro* by incorporating Si in the culture medium. However, further studies required to evaluate the effect of different source and concentration of silicon on the growth and development of various plants.

## ABIOTIC STRESS TOLERANCE

Duan et al. (2013) reported that Si enhance cold resistance of *Dendrobium moniliforme* by increasing the content of free proline, soluble sugar and soluble protein and decreasing MDA content. Si treatment improved the survival rate of grape ‘Kyoho’ and ‘koshusanjaku’ calli under low temperature by preventing browning (Moriguchi et al., 1988). *In vitro* storage of *Coleus hybridus ‘*Jupiter’ and *Solanum tuberosum* var. *Gersa* under silicone oil significantly reduced the growth and maintained their regenerative potential (Radovet et al., 2008; Radovet-Salinschi and Cachita-Cosma, 2012). These results reveal that Si can be used as cryoprotectant and included in the cryoprotective mixture for minimizing the toxicity of cryoprotectants. The ameliorating effect of Si on salt stress *in vitro* has been reported in *A. multiflora* (Sivanesan and Jeong, 2014), *Salvia splendens* ‘Hot Jazz’ (Soundararajan et al., 2013) and *S. tuberosum* (Qing et al., 2005). Si alleviates salt stress in plants by limiting NaCl uptake, maintenance of ultrastructure of stomata, improving photosynthetic activity, reducing free proline content and altering the production of antioxidant enzymes (Qing et al., 2005; Soundararajan et al., 2013; Sivanesan and Jeong, 2014). Prabagar et al. (2011) investigated the effect of Si on aluminium (Al) tolerance in *Picea abies* suspension cultures. Al toxicity was reduced when the liquid medium was supplemented with Si and the effect was increased at pH 5.0 than pH 4.2. Si supplementation protected *P. abies* cells and against Al toxicity by reducing the concentration of free Al in the cell wall. Si is also reported to enhance drought tolerance, alleviate lead toxicity and increase resistance to radiation and temperature stresses (Balakhnina and Borkowska, 2013; Zhu and Gong, 2014). The molecular mechanisms of Si on stress tolerance are poorly understood. Thus, more studies are needed to find out the role of Si in abiotic tolerance on various plants.

## FUTURE PROSPECTS

Recent studies have shown the beneficial effects of Si in plant tissue culture (**Table 1**). However, further studies on a wide variety of plant species are needed to confirm the role of Si in plant tissue culture. *In vitro* culture is a useful system for studying physiological and biochemical functions of Si in plants at molecular level. Further, *in vitro* cell suspension culture systems provide an opportunity to study roles of Si at the single cell level. The inclusion of silicone A to Linsmaier and Skoog liquid medium also enhances cell growth and anthocyanins content in cell suspension culture of *Perilla frutescens* (Zhong et al., 1992). Thus, Si can also be used for the stimulation of secondary metabolites in the plant cell, tissue and organ cultures. We strongly recommend the inclusion of Si as a beneficial nutrient in the tissue culture medium to solve various micropropagation problems, and to increase tissue culture success.

## Conflict of Interest Statement

The authors declare that the research was conducted in the absence of any commercial or financial relationships that could be construed as a potential conflict of interest.
